# Standardized template for clinical reporting of PSMA PET/CT scans

**DOI:** 10.1007/s00259-024-06857-w

**Published:** 2024-08-15

**Authors:** Shadi A. Esfahani, Michael J. Morris, Oliver Sartor, Mark Frydenberg, Stefano Fanti, Jeremie Calais, Neha Vapiwala

**Affiliations:** 1 Division of Nuclear Medicine and Molecular Imaging, Department of Radiology, Massachusetts General Hospital, Harvard Medical School, Boston, MA USA; 2 Genitourinary Oncology Service, Department of Medicine, Memorial Sloan Kettering Cancer Center, New York, NY USA; 3 Department of Medical Oncology, Mayo Clinic, Minnesota, USA; 4 Department of Surgery, Faculty of Medicine, Nursing and Health Sciences, Monash University, Melbourne, Australia; 5 Cabrini Institute, Cabrini Health, Malvern, Australia; 6 IRCCS Azienda Ospedaliero-Universitaria di Bologna, Bologna, Italy; 7 Ahmanson Translational Theranostics Division, Department of Molecular and Medical Pharmacology, David Geffen School of Medicine at UCLA, University of California, Los Angeles, CA USA; 8 Department of Radiation Oncology, University of Pennsylvania, Philadelphia, PA USA

**Keywords:** PSMA, PET/CT, Report, Prostate cancer, Guideline

## Abstract

**Purpose:**

Accurate diagnosis and staging of prostate cancer are crucial to improving patient care. Prostate-specific membrane antigen (PSMA)-targeted positron emission tomography with computed tomography (PET/CT) imaging has demonstrated superiority for initial staging and restaging in patients with prostate cancer. Referring physicians and PET/CT readers must agree on a consistent communication method and application of information derived from this imaging modality. While several guidelines have been published, a single PSMA PET/CT reporting template has yet to be widely adopted. Based on the consensus from community and academic physicians, we developed a standardized PSMA PET/CT reporting template for radiologists and nuclear medicine physicians to report and relay key imaging findings to referring physicians. The aim was to improve the quality, clarity, and utility of imaging results reporting to facilitate patient management decisions.

**Methods:**

Based on community and expert consensus, we developed a standardized PSMA PET/CT reporting template to deliver key imaging findings to referring clinicians.

**Results:**

Core category components proposed include a summary of any prior treatment history; presence, location, and degree of PSMA radiopharmaceutical uptake in primary and/or metastatic tumor(s), lesions with no uptake, and incidentally found lesions with positive uptake on PET/CT.

**Conclusions:**

This article provides recommendations on best practices for standardized reporting of PSMA PET/CT imaging. The generated reporting template is a proposed supplement designed to educate and improve data communication between imaging experts and referring physicians.

**Supplementary Information:**

The online version contains supplementary material available at 10.1007/s00259-024-06857-w.

## Background

### Prostate-specific membrane antigen

Prostate cancer (PCa) is the most diagnosed cancer among males worldwide and the accurate methods for diagnosis, staging, and restaging of this disease are crucial for patient management. Prostate-specific membrane antigen (PSMA) positron emission tomography (PET) scan has recently been adopted as an imaging modality for men with PCa. PSMA is a membrane-bound metallopeptidase glycoprotein encoded by the folate hydrolase 1 gene on chromosome 11 [1]. It is expressed in several tissues with moderate to intense physiological uptake seen in the liver, duodenum, parotid glands, ganglions, and others [2, 3] (Supplemental Table 1). Additionally, PSMA is overexpressed in over 90% of PCa cells. In untreated patients, PSMA overexpression increases with the tumor grade and aggressiveness [4].

PSMA-targeting compounds (e.g., PSMA-11, PSMA I&T, PSMA-617, PSMA-1007, DCFPyL, rhPSMA7.3) can be coupled with positron emitting radioisotopes such as gallium-68, fluorine-18 or copper-64 to form a PET radiopharmaceutical [5]. Following injection, the PSMA-targeted radiopharmaceutical rapidly clears out from the bloodstream, binds to the PSMA site, and gets internalized via clathrin-coated pits and then endocytosed [6]. Because of the typically high density of PSMA on the surface of PCa cells relative to the adjacent prostate (for primary tumor) or other close or distant non-neoplastic background tissues (for primary and metastatic tumors), PSMA PET provides images with a high tumor-to-background uptake ratio [7–9]. PSMA PET has been shown to have higher sensitivity and specificity than conventional imaging for PCa in several clinical settings, from initial staging to detection and localization of biochemical recurrence (BCR), restaging and assessment of eligibility for PSMA-targeted radiopharmaceutical therapies [10].

PSMA positron emission tomography and computed tomography (PET/CT) imaging is increasingly adopted in routine clinical practice worldwide [11]. Developing consensus guidelines for interpreting PSMA PET may improve the quality of care by reducing variability in interpretation through accurate quantification of disease burden with increased reproducibility on a patient level, which may lead to more accurate diagnoses and appropriate treatment strategies. Moreover, standardized reporting facilitates clearer communication among the multidisciplinary team members involved in PCa care, including urologists, oncologists, radiation oncologists, and pathologists. This can lead to more effective and coordinated patient management [12]. The currently available guidelines are directed more towards academic imaging experts, and their terminology and technical details may be less familiar or useful to the community imaging experts and clinicians. In this paper, we propose a practical, downloadable, standardized PSMA image reporting tool targeted towards community physicians that includes information for the referring providers to understand a patient’s full clinical picture and to better help determine patient management [13].

### Clinical utility of PSMA

Most primary and metastatic prostate adenocarcinomas demonstrate PSMA overexpression, which correlates with disease aggressiveness in the treatment naïve setting [14]. The use of PSMA PET/CT is increasing in routine clinical practice, such as in the initial staging of high-risk primary PCa, patients with BCR, follow-up during and after local or systemic treatment, and, recently, several centers use it for intraprostatic evaluation of the lesions where magnetic resonance imaging (MRI) is contraindicated and for assessment of extra-prostatic extension for surgical guidance in PCa [15–17]. The adoption of PSMA PET/CT has resulted in a profound transformation of PCa management since its FDA approval and addition to various international cancer management guidelines (Supplemental Table 2) in recent years [13, 18–23].

Despite the “prostate-specific” term, PSMA is also expressed in non-prostatic tissues and other pathologic conditions and can be visualized through the urinary, salivary, or hepatobiliary system where it is physiologically excreted [24]. In addition, PSMA is expressed on neovascular cells, other neoplasms, and inflammatory or remodeling processes [25, 26]. Different radiopharmaceuticals exhibit unique physiological distribution patterns [27, 28]. Reader training and knowledge of the normal biodistribution of PSMA-targeted radiopharmaceutical and understanding other pathological conditions with increased radiopharmaceutical uptake on PET/CT are important for precise interpretation [2, 15, 24, 29]. One study reported that properly trained physicians accurately interpret [^68^Ga]Ga-PSMA-11 PET images in up to 90% of cases [30].

### Gaps and barriers in current reporting guidelines

Risk stratification is an important factor for deciding the further course of management following diagnosis. Currently, this is based on various clinical and histopathological factors and predictive mathematical models, as seen in the D’Amico, National Comprehensive Cancer Network^®^, and University of California San Francisco-Cancer of the Prostate Risk Assessment risk stratifications [31, 32]. The commonly used variables are prostate-specific antigen (PSA), Gleason score, and clinical T/N/M stage to divide patients into now practiced three risk categories of low, intermediate, and high risk [32]. Thus, PSMA PET/CT can help direct risk stratification and monitoring by more accurately identifying localized tumors (T), nodal metastases (N), and distal metastases (M) for clinician management [33]. PSMA PET imaging of PCa has potential equivocal findings and interpretive pitfalls, as with any other imaging test [34].

Currently, several reporting guidelines have been suggested (Prostate Cancer Molecular Imaging Standardized Evaluation [PROMISE], Prostate-specific Membrane Antigen Reporting and Data System [PSMA-RADS], European Association of Nuclear Medicine-Prostate-Specific Membrane Antigen [E-PSMA]), which vary in reporting details; however, none are utilized in the community practice setting (Supplemental Table 3) [2, 34, 35]. The reasons for this lack of utilization in the community may include:

1. None of the current reporting guidelines developed have been officially adopted among radiological or clinical societies and, as a result, their use has been largely limited to imaging experts in academic circles or clinical research.
2. With no single source, a number of different PSMA imaging guidelines have been created and are available; moreover, the volume of information within each guideline is extensive and can be difficult to navigate.
3. The available guidelines are directed more towards academic imaging experts, and their terminology and technical details may be less familiar or useful to the community imaging experts and clinicians. Moreover, community radiologists can often face greater time pressures due to higher patient loads and may prioritize the need to maximize throughput over implementing an additional protocol.

Nonetheless, community physicians, including imaging experts and referring providers, would greatly benefit from disseminating a standardized, easily communicated reporting template as the PSMA technology grows and becomes increasingly applicable to the clinical management of patients.

To this end, we developed a practical, multidisciplinary, and downloadable template for PSMA image reporting to overcome these barriers and augment the implementation of a standardized PSMA imaging guideline within the community setting. Moreover, unlike the other templates, this template was developed with the input of an expert panel that includes the input from community radiologists in order to properly hone the most clinically relevant reporting information in a community clinical setting.

## Methods

An independent, multidisciplinary panel of global expert physicians involved in PCa patient care (including radiologists, medical oncologists, nuclear medicine physicians, radiation oncologists and urologists from both academic institutions and community practices) convened throughout several meetings to discuss current reporting practices across institutions and countries. Panel participants identified the gaps and implementation of PSMA PET guidelines to aid the understanding of current practices across settings. Common themes, data elements, and their relationship to prognostication and relevance to treatment consideration were identified, from which a concise set of reporting criteria deemed most relevant across a multidisciplinary platform intended to aid patient management decisions was agreed upon in the context of PSMA PET/CT imaging. The main output was the development of an easily implementable, user-friendly, standard PSMA PET/CT reporting template with the minimum necessary information, for radiologists and nuclear medicine physicians to provide to referring physicians. Results from panel participant discussions are presented.

## Results

### Template components

#### Clinical history & procedure

**Fig. 1:**
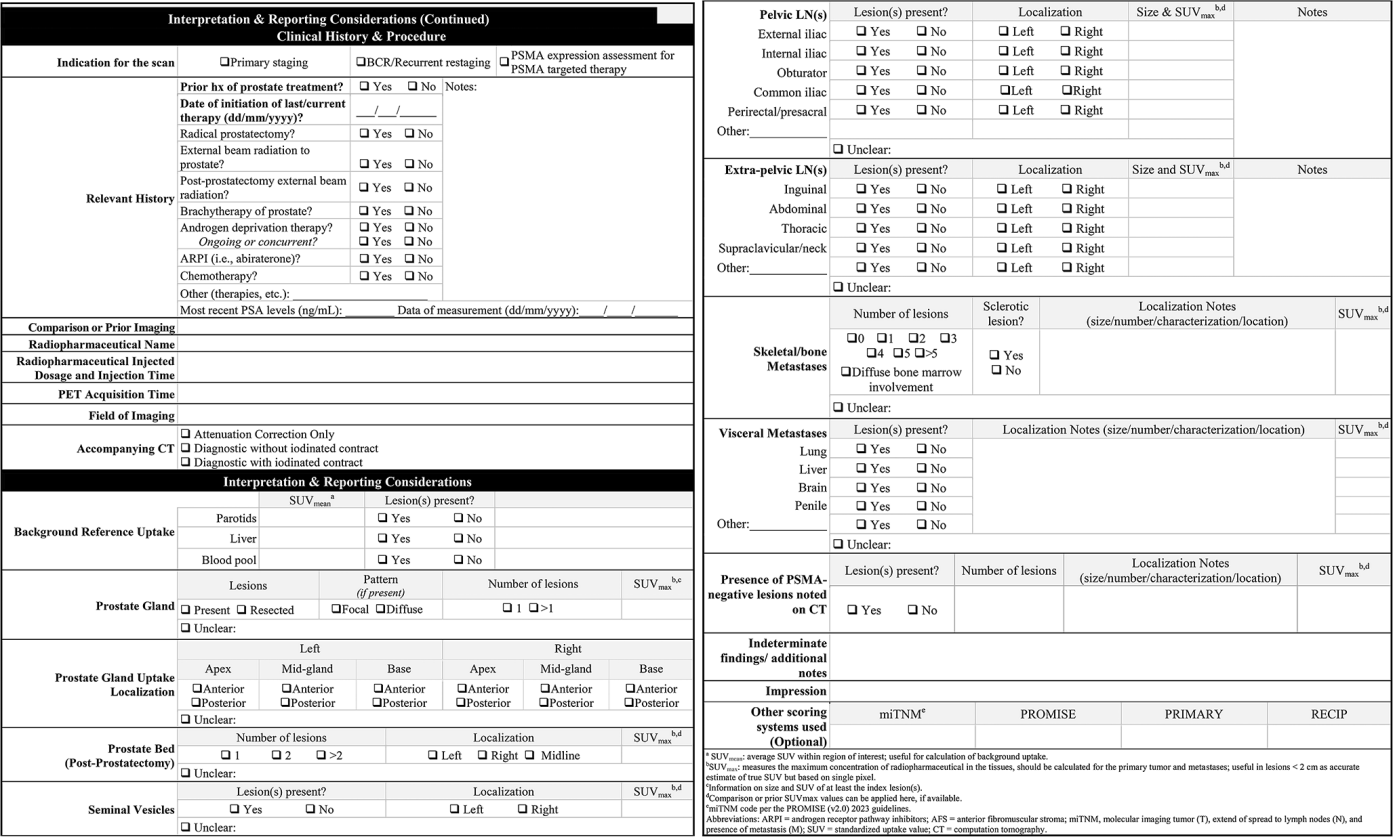
PSMA PET/CT image reporting template

Panel participants agreed the imaging report should begin with a standardized description of pertinent clinical history, similar to other existing guidelines. Clinical history should include the diagnosis and reason(s) for the referral (primary staging, BCR/ restaging, or PSMA target expression assessment for PSMA-targeted therapy). Relevant oncological history is an essential aspect when reviewing each patient and to minimize the need for freehand text, checkboxes of common treatment options were incorporated to facilitate rapid review. A comparison or prior PSMA PET/CT scans (if available) is critical for assessing the progression or regression of disease overtime and included in the template. Date of initiation of ongoing or concurrent therapy with type of therapy should be noted. The results of relevant diagnostic tests, especially PSA level, should be summarized. Procedural and technical details should include the type and dosage of radiopharmaceutical, injection time, PET acquisition time and field of imaging (Fig. 1). Imaging acquisition should begin at the proximal thighs and proceed cranially to skull base or vertex.

#### Interpretation & reporting considerations

Since normal and variable PSMA-targeted radiopharmaceutical uptake can be found in lacrimal/salivary glands, liver, spleen, kidney, digestive tract, ureters, and bladder [2, 24], imaging experts should consider potential false positive findings where increased PSMA uptake is considered normal. In addition, the radiopharmaceutical uptake level relative to the background is important in patients that may be candidates for systemic PSMA-targeted radiopharmaceutical therapy [36]. Thus, background reference uptake for parotid glands, liver, and blood pool was included as part of this image reporting template.

In the general review, attention should be paid to the presence of lesions and, if present, their size, location, and SUVmax; regions that may relate to any symptoms or pathology noted on the referral form should be given specific attention. The panel agreed that emphasis should be placed on the prostate gland/bed, seminal vesicles, pelvic lymph nodes (including external iliac, obturator, internal iliac, common iliac, and perirectal/presacral), and extra-pelvic lymph nodes (including abdominal, thoracic, supraclavicular/neck, and inguinal). Common locations for potential metastases are similarly important to decision patient management and thus, similar data should be recorded for skeletal system and visceral metastases (such as lung, liver, brain, and penis) found. These inclusion categories will similarly help direct risk stratification and monitoring by more accurately identifying localized tumors (T), nodal metastases (N), and distal metastases (M) for clinician management. The panel agreed that the presence of PSMA-negative findings and additional PSMA-positive lesions should be reported if detected on CT scan. Lastly, indeterminate findings or additional notes should be included to encompass other image reporting not represented in this template, including the option to include the miTNM code per the PROMISE (v2.0) 2023 guidelines, PROMISE, PRIMARY, and RECIP guidelines [35]. An infographic with instructions on how to approach this PSMA image reporting template, including detailed information on the purpose of each section, can be seen in Supplemental Fig. 1.

### Limitations

Despite the significant advantages and clinical utility of the standardized PSMA PET/CT reporting template developed in this study, several potential limitations and sources of error should be acknowledged:

- The challenge of variability in interpreting PSMA PET/CT scans persists, primarily due to differences in reader experience and expertise. Even with standardized reporting guidelines, the potential for discrepancies in the subjective assessment of PSMA-targeted radiopharmaceutical uptake and lesion characterization remains, which could significantly influence clinical decision-making.
- The inherent biological variability in PSMA expression among different patients and within different tumor sites can lead to variability in imaging results, which may affect the reproducibility and accuracy of the scans.
- There is always a potential for false-positive or false-negative findings due to the physiological uptake of PSMA-targeted radiopharmaceutical in non-prostatic tissues and benign conditions, which can be mistaken for pathological uptake.
- The study’s reliance on expert consensus may introduce biases related to the experts’ specific clinical practices and interpretations, which might only partially represent the broader clinical community.
- The suggested template has not yet been tested in a real-world clinical setting, which may reveal unforeseen issues and further areas for improvement.
- Implementing this standardized template in routine clinical practice may require more support, such as additional training and potential resistance to change from established reporting practices.

It is important to address these limitations through continuous education, validation studies, and iterative refinement of the guidelines to ensure the effectiveness and broad adoption of the standardized PSMA PET/CT reporting template.

## Conclusion

PSMA PET/CT is an imaging modality for PCa that can be indicated for primary staging, restaging, targeted biopsies, evaluation for PSMA-targeted therapies and monitoring treatment response in PCa. Several reporting guidelines have been developed and vary in reporting details; however, none have been officially adopted among radiological or clinical societies and are wholly underutilized by community imaging experts and referring physicians. Here, we have provided a high-level overview of PSMA PET/CT imaging and guidelines, along with the development of a standardized reporting tool by a panel of experts composing both academic and community physicians. This reporting tool is intended to complement the existing international guidelines, help understand the patient’s full clinical picture, and facilitate overall patient management. This article extends the existing body of standardized reporting guidelines for PSMA PET/CT imaging aiming at improving the clarity and quality of reporting, decreasing the ambiguity in communication of findings, and increasing precision, repeatability, and utility of the clinical decision report.

## Electronic supplementary material

Below is the link to the electronic supplementary material.

Supplementary Material 1

## Data Availability

The original contributions presented in the study are included in the article. Further enquiries can be directed to the corresponding author.
