# Supplementary material for: Standardized template for clinical reporting of PSMA PET/CT scans
Source: Eur J Nucl Med Mol Imaging. 2024 Aug 15;52(1):335–41. doi: 10.1007/s00259-024-06857-w (PMC11599343; doi:10.1007/s00259-024-06857-w)
Supplement: Supplementary file 1 — Supplementary Material 1 [file 259_2024_6857_MOESM1_ESM.docx]

**SUPPLEMENTAL DOCUMENT**

**Supplemental** **Table 1**:
Normal Physiologic Distribution of PSMA-PET Radiopharmaceuticals …..…………………………………………..………2

**Supplemental Table 2**:
Current Summary of Recommendations on Use of PSMA PET (PET/CT, PET/MRI) Imaging by Guideline………..……….3

**Supplemental Table 3**:
Summary of Currently Available PSMA PET Reporting………………………………………………………………………4

**Supplemental Figure 1:**Instructions on how to approach PSMA Image Reporting Template……….………………………………………………….5

| **Supplemental Table 1**^2, 3^ Normal Physiologic Distribution of PSMA-PET Radiopharmaceuticals | | |
| --- | --- | --- |
| **Variable - Moderate** | **Moderate-to-High** | **High** |
| Oropharyngeal, laryngeal, and esophageal mucosa* | Duodenum^†^ | Kidneys, ureters, and bladder |
| Liver | Bowel | Salivary glands^§^ |
| Spleen |  | Lacrimal gland |
| Testes |  |  |
| Sympathetic ganglia^‡^ |  |  |
| *Due to salivary excretion  ^†^PSMA expression may facilitate absorption of dietary folates ^‡^Including celiac, stellate, hypogastric, and presacral ganglia  ^§^Including parotid and submandibular  PSMA = prostate-specific membrane antigen; PET = positron emission tomography. | | |

**Supplemental Table 2**Current Summary of Recommendations on Use of PSMA PET (PET/CT, PET/MRI) Imaging by Guideline

| **CLINICAL STAGE** | | **EAU^18^** | **ASCO^19, 20^** | **EANM/SNMMI v2.0^13^** | **NCCN v1.2023^21^** | **RADAR VI TPI^22^** | **AUA/ASTRO^23^** |
| --- | --- | --- | --- | --- | --- | --- | --- |
| Diagnosis | | Not recommended | Not recommended | N/A | Not recommended |  | Not recommended |
| Staging | | PSMA PET/CT; not recommended; perform at least cross-sectional imaging for intermediate and high-risk patients | When conventional imaging is negative in patients with high risk of metastatic disease, NGI may add clinical benefit, although prospective data are limited; when conventional imaging is suggestive or equivocal, NGI may be offered to patients for clarification of equivocal findings or detection of additional sites of disease, which could potentially alter management, although prospective data are limited | Recommendation of use for initial staging of unfavorable intermediate  to high‑risk prostate cancer | For symptomatic patients and/or those with a life expectancy of greater than 5 years, bone and soft tissue imaging is appropriate; [^68^Ga]Ga-PSMA-11 or [^18^F]F-DCFPyL PET/CT or PET/MRI (full body imaging) can be considered as an alternative to bone scan | TPI (including [^18^F]F-fluciclovine, [^68^Ga]Ga-PSMA, and [^18^F]F-DCFPyL PET/CT) is recommending for:   - Unfavorable intermediate-risk, high-risk, or very high-risk PCa ± CI - Inconclusive prior CI in which there is a high clinical suspicion of metastatic or locoregional disease or risk of nodal metastasis - Patients who have an elevated molecular marker score - Patient populations with genomically/diverse high-risk | Not recommended |
| BCR | | Perform PSMA PET/CT if PSA>0.2 ng/mL post-radical prostatectomy and if results will influence subsequent treatment decisions | Goal of therapy and potential use of salvage local therapies in these scenarios should guide choice of imaging | Recommendation of use for localization of recurrent (BCR) or persistent prostate cancer following curative‑intent therapy | Because of the increased sensitivity and specificity of PSMA-PET tracers for detecting micrometastatic disease compared to conventional imaging (CT, MRI) at both initial staging and biochemical recurrence, the panel does not feel that conventional imaging is a necessary prerequisite to PSMA-PET and that PSMAPET/CT or PSMA-PET/MRI can serve as an equally effective, if not more effective front-line imaging tool for these patients(52) | Consider TPI in patients with rising PSA at or above 0.2 ng/dL for:   - The patient for whom MDT may be considered, TPI is preferred over CI - Early and accurate identification of sites of disease can lead to more anatomically directed therapy - Optimizing outcomes can be facilitated by early detection of the site(s) of recurrent disease | N/A |
| PSA persistence | | Offer PSMA PET/CT to men with persistent PSA. 0.2 ng/mL post-surgery to exclude metastatic disease | For men for whom salvage local or regional therapy is contemplated, there is evidence supporting NGI for detection of local or distant sites of disease |  |  |  | N/A |
| Advanced PCa | Nonmetastatic CRPC | With more sensitive imaging techniques such as PSMA PET/CT or whole-body MRI, more patients are expected to be diagnosed with early metastatic CRPC | For men with nonmetastatic CRPC, NGI can be offered only if change in clinical care is contemplated | Recommendation of use for localization of castrate resistant prostate cancer which is non‑metastatic by conventional imaging; may aid patient stratification in clinical trials and adds information for therapy guidance |  | TPI appropriate use criteria:   - Patients whose disease is detectable only by TPI and not by CI should generally be treated as M0 CRPC - Role of TPI while on therapy for M0 CRPC who had baseline imaging:   *Systemic therapy*: Imaging should be repeated at least annually and at the time of PSA recurrence.  *Local therapy*: TPI may be repeated within 4-6 mo to document the response to therapy and assess for new metastases and then repeated annually as above | In patients with prostate cancer at high risk for metastatic disease with negative conventional imaging, clinicians may obtain molecular imaging (including PSMA) to evaluate for metastases. |
|  | Metastatic CRPC | Use of PSMA PET/ CT scans for progressing CRPC is not established | Use of NGI in this cohort is unclear, with paucity of prospective data. When a change in clinical care is contemplated and there is a high clinical suspicion of subclinical metastasis despite negative conventional imaging, the use of NGI could be contemplated. In clear evidence of radiographic progression on clinical investigation, NGI should not be routinely offered. | Recommendation of use for staging before PSMA‑directed RLT for metastatic prostate cancer | Bone imaging can be achieved by conventional [^99m^Tc]Tc-MDP bone scan. Plain films, CT, MRI, or PET/CT or PET/MRI with [^18^F]F-sodium fluoride, [^11^C]C-choline, [^18^F]F-fluciclovine, [^68^Ga]Ga-PSMA-11, or [^18^F]F-DCFPyL can be considered for equivocal results on initial bone imaging. | - *Initial staging*: TPI is recommended for staging of M1 CRPC if it is likely to affect clinical decisions - *Treatment monitoring:* At present, response criteria are lacking for TPI. TPI should not be used alone to make decisions regarding treatment discontinuation in the context of M1 CRPC. | N/A |
| PSMA targeted therapy | | N/A | N/A | PSMA-ligand PET/CT can be performed in patients with advanced prostate cancer to confirm eligibility for targeted therapy and to assess the likelihood of response to targeted therapy. No specific recommendation for the use of PSMA targeted therapy is provided. | Either [^68^Ga]Ga-PSMA-11 or F- [^18^F]F-DCFPyL imaging can be used to determine eligibility. | *Treatment eligibility*: PSMA-PET imaging can be used for treatment selection for PSMA-targeted therapies. | N/A |
| ASCO = American Society of Clinical Oncology; BCR = biochemical recurrence; CI = conventional imaging; CRPC = castrate-resistant prostate cancer; CT = computed tomography; EANM = European Association of Nuclear Medicine; EAU = European Association of Urology; MDT = metastasis-directed therapy; NCCN = National Comprehensive Cancer Network^®^; NGI = next generation imaging*; PET = positron emission tomography; PSA = prostate specific antigen; PSMA = prostate-specific membrane antigen; SNMMI = Society of Nuclear Medicine and Molecular Imaging; TPI = targeted precision imaging.  *Defined as PET, PET/CT, PET/MRI, whole-body MRI | | | | | | | |

**Supplemental Table 3**
Summary of Currently Available PSMA PET Reporting

| **GUIDELINE**  **(YEAR)** | **SUMMARY** | **PSMA PET READING SCORE** | | **PSMA PET INTERPRETATION SCORE** | |
| --- | --- | --- | --- | --- | --- |
| **PROMISE**^35^ (2023) | The PROMISE criteria are a suggested standardization for PSMA PET/CT both for reading based on the intensity of PSMA expression (miPSMA scores) and for interpretating the images and staging the disease (miTNM scores). The miTNM score organizes the staging of whole-body prostate cancer by including information on exact location, pattern of disease distribution, PSMA expression, and level of certainty. | **miTNM scores^†^** | | **Class** | **Description** |
|  |  | Local tumor (T) | | miT0 | No local tumor |
|  |  |  |  | miT2 | Organ-confined tumor (unifocally versus multifocality) |
|  |  |  |  | miT3a | Non-organ-confined tumor (extracapsular extension) |
|  |  |  |  | miT3b | Tumor invades seminal vesicle (s) |
|  |  |  |  | miT4 | Tumor invading adjacent structures other than seminal vesicles, such as external sphincter, rectum, bladder, levator muscles, and/or pelvic wall |
|  |  |  |  | miTr | Presence of local recurrence after radical prostatectomy |
|  |  | Intrapelvic nodes (N) | | miN0 | No positive regional lymph nodes |
|  |  |  |  | miN1 | Single lymph node region harbors lymph node metastases, report location by a standardized template (lymph node regions with laterality [left/right]: internal iliac, external iliac, obturator, presacral, other pelvic) |
|  |  |  |  | miN2 | Multiple (≥2) lymph node regions harbor lymph node metastases, report location(s) by a standardized template |
|  |  | Distant metastases (M)^‡^ | | miM0 | No distant metastases |
|  |  |  |  | miM1a | Distant lymph node region(s) (common iliac, retroperitoneal, supradiaphragmatic, inguinal and other extrapelvic) |
|  |  |  |  | miM1b | Bone(s), additionally report pattern and involved bone(s) in case of unifocal or oligometastatic (uptake patterns: unifocal, oligometastatic, disseminated, diffuse marrow involvement) |
|  |  |  |  | miM1c | Other site(s), additionally report involved organ (hepatic, pulmonary, adrenal, brain, other). Other includes pleural or peritoneal invasion |
| **PSMA-RADS Version 1.0**^34^  (2018) | PSMA-RADS is a framework for classifying PSMA-targeted PET scans and individual findings on these studies into categories that reflect the likelihood of the presence of PCa. The system is optimized for findings outside of the prostate and is not intended to replace PI-RADS for categorizing findings on prostate MRI(69) | PSMA-RADS-1 (*benign*) | -1A | Benign lesion characterized by biopsy or pathognomonic finding on anatomic imaging and without abnormal uptake | |
|  |  |  | -1B | Benign lesion characterized by biopsy or pathognomonic finding on anatomic imaging and with focal radiotracer uptake | |
|  |  | PSMA-RADS-2  (*likely* *benign*) | | Equivocal uptake in soft-tissue site atypical of PCa involvement; equivocal uptake in bone lesion atypical of PCa involvement | |
|  |  | PSMA-RADS-3 (*equivocal**) | -3A | Equivocal uptake in soft-tissue site typical of PCa involvement. If targetable for biopsy, may help confirm diagnosis. | |
|  |  |  | -3B | Equivocal uptake in bone lesion not definitive but also not atypical of PCa on anatomic imaging. Comparison to bone scan, Na18F PET, or tumor-protocol MRO images may be helpful, and bone biopsy may have a role. | |
|  |  |  | -3C | Intense uptake in site is highly atypical of all but advanced stages of PCa. Likelihood of nonprostatic malignancy or other benign tumor is high. | |
|  |  |  | -3D | Lesion suggestive of malignancy on anatomic imaging but lacking uptake. Differential considerations include nonprostatic malignancy, neuroendocrine PCa, and an uncommon case of prostate adenocarcinoma that fails to express PSMA. | |
|  |  | PSMA-RADS-4  (*PCa highly likely*) | | Intense uptake in site typical of PCa but lacking definitive findings on conventional imaging. | |
|  |  | PSMA-RADS-5  (*PCa almost certainly present*) | | Intense uptake in site typical of PCa and having corresponding findings on conventional imaging. | |
| **E-PSMA v1.0**^2^  (2021) | The E-PSMA is a comprehensive guideline supported by the European Association of Nuclear Medicine and aims to develop a structured report for PSMA PET images and to harmonize diagnostic interpretation criteria. In the suggested structured EPSMA report, the visual description is adapted from PROMISE guidelines. For image interpretation, the guideline panelists suggest a 5-point scale of confidence, shown here. | E-PSMA scores* | |  | |
|  |  | 1 | | Benign lesion without abnormal PSMA uptake | |
|  |  | 2 | | Probably benign lesion: faint PSMA uptake (equal or lower than background) in a site atypical for prostate cancer | |
|  |  | 3 | | Equivocal finding: faint uptake in a site typical for prostate cancer or intense uptake in a site atypical for prostate cancer | |
|  |  | 4 | | Probably prostate cancer: intense uptake in typical site of prostate cancer, but without definitive findings on CT^∥^ | |
|  |  | 5 | | Definitive evidence of prostate cancer: intense uptake in typical site of prostate cancer, with definitive findings on CT | |
| PSMA = prostate-specific membrane antigen; RADS = reporting and Data system; PCa = prostate cancer; MRI, molecular resonance imagine; PET = positron emission tomography; CT = computer tomography.  ^†^Designed mainly for [^68^Ga]Ga-PSMA-11. For PSMA ligands with liver-dominant excretion, spleen is recommended as reference organ instead of liver.  ^‡^Scores 2 and 3 should be considered PCa lesions, depending on clinical context, extent, and location of findings  ^§^Designed for most extensively used PSMA ligands ([^68^Ga]Ga-PSMA-11, [^18^F]F-DCFPyL, and [^18^F]F-PSMA-1007). ^∥^A definitive finding on CT means the presence of a real anatomical substrate on the CT. | | | | | |

**Supplemental Figure 1**Instructions on how to approach PSMA Image Reporting Template

**Purpose**: Helps in tailoring the interpretation of the scan to the specific clinical scenario, ensuring that the radiologist's insights are directly relevant to the patient's current health status and treatment plan.

Additional relevant information not described in the list (left) should be in this notes section.


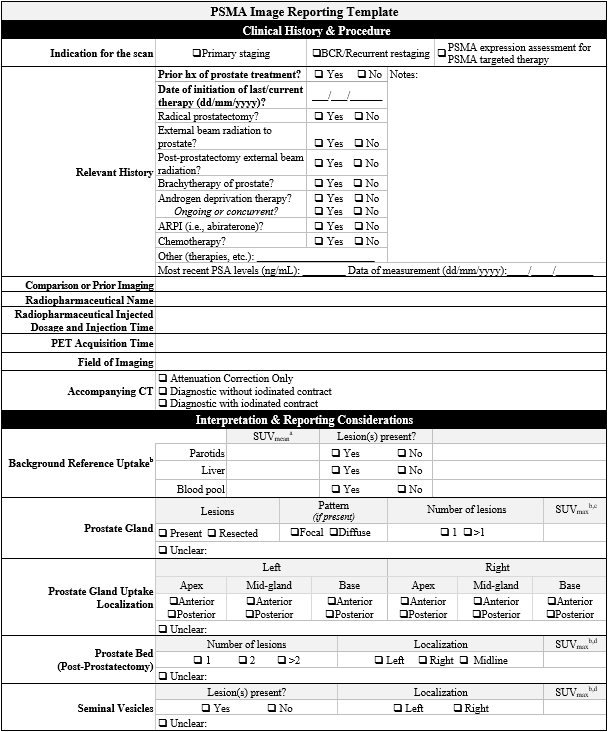


**Purpose:** Provides a detailed account of the patient's cancer history, including the type and stage of prostate cancer, previous treatments (surgery, radiation, hormonal therapy), and outcomes of these treatments.


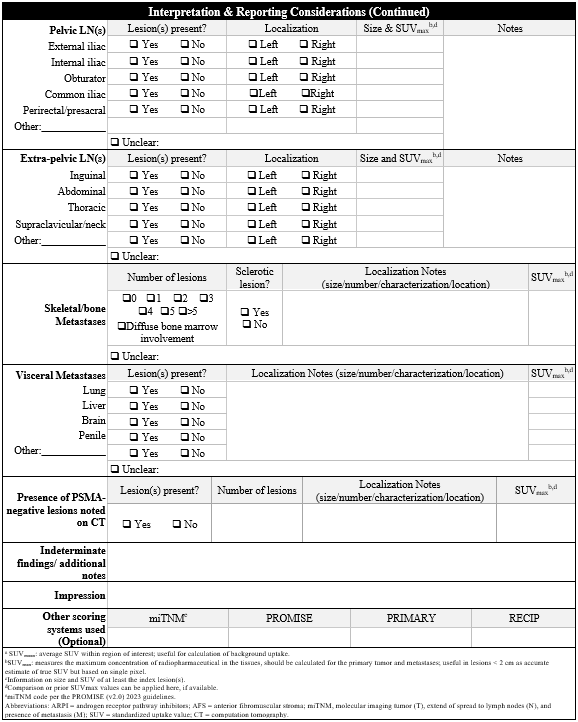


**Purpose:** Provides space for documenting findings that are unclear or require further investigation, or observations that do not fit into the standard categories.

**Purpose:** Identifies lesions that are visible on CT but do not exhibit PSMA uptake, which could indicate non-prostate cancer pathology or PSMA-negative prostate cancer. Important for a comprehensive assessment and to avoid missing significant pathology.

**Purpose:** Describes any metastatic lesions in organs such as the liver, lungs, or brain, including their size and location. Essential for comprehensive staging, as visceral metastases can significantly impact treatment decisions and prognosis.

**Purpose:** Records the number, type (e.g., sclerotic or lytic), and locations of bone metastases, which are common in advanced prostate cancer. This information is vital for assessing the extent of metastatic disease, planning treatment, and monitoring response to therapy.

**Purpose:** Documents the presence and characteristics of metastatic lesions in lymph nodes outside the pelvic area, providing crucial information for staging and assessing the overall burden of the disease. Helps in identifying the extent of metastatic spread, which is critical for determining the appropriate systemic therapy.

**Purpose:** Details the presence of any lesions in the pelvic lymph nodes, specifying their exact anatomical location and size. This is crucial for accurate staging, particularly in determining the N (nodal) stage in the TNM classification system for prostate cancer.

**Purpose:** The SUV_max_ provides insight into the metabolic activity of these lesions, which can guide further management, including the need for additional local therapy or systemic treatment.

**Purpose:** Provides baseline uptake values for comparison, helping to distinguish between normal physiological uptake and pathological uptake indicative of disease. Assists in identifying potential false positives by establishing expected uptake levels in non-target tissues. Assists with identifying lesions with uptake above background.

**Purpose:** Identifies the specific PSMA radiotracer used, as different agents may have varying affinities and specificities, impacting the interpretation of the scan.

**Purpose**: Records the exact dose of the radiotracer administered and the precise time of its administration, as variations can affect the intensity and distribution of radiotracer uptake. Critical for standardizing imaging protocols and ensuring comparability between different scans and patients.

**Purpose:** Important for ensuring that the scan is performed during the optimal window for imaging PSMA expression in prostate cancer cells.

**Purpose:** If available, this comparison is critical for assessing the progression or regression of disease over time.

**Purpose:** Specifies the anatomical range covered by the scan, which is crucial for ensuring that all relevant areas (e.g., pelvic region, abdomen, thorax) are included for a comprehensive assessment. Consistency in the field of imaging is key for comparing scans over time and between different patients.

**Purpose:** Reports on the presence and characteristics of lesions in the seminal vesicles and pelvic lymph nodes, including their metabolic activity, which is crucial for staging and determining the extent of local spread.

**Purpose:** Reports on the presence and characteristics of lesions in the prostate bed post-prostatectomy. This information is crucial for detecting local recurrence, which is a key consideration in post-prostatectomy patients.

**Purpose:** Describes the precise areas within the prostate gland showing increased PSMA uptake, which can indicate the primary site of the tumor and potential multifocal disease. This information is vital for guiding biopsies, surgical planning, and targeted therapies.

**Purpose:** Details the characteristics of any lesions within the prostate. These metrics are crucial for assessing the primary tumor and planning local treatment strategies.
